# A rice small GTPase, Rab6a, is involved in the regulation of grain yield and iron nutrition in response to CO_2_ enrichment

**DOI:** 10.1093/jxb/eraa279

**Published:** 2020-06-11

**Authors:** An Yang, Qian Li, Lei Chen, Wen-Hao Zhang

**Affiliations:** 1 State Key Laboratory of Vegetation and Environmental Change, Institute of Botany, the Chinese Academy of Sciences, Beijing, China; 2 College of Horticulture, Northwest A&F University, Yangling, Shaanxi, China; 3 Guangdong Provincial Key Laboratory of Biotechnology for Plant Development, School of Life Sciences, South China Normal University, Guangzhou, China; 4 College of Resources and Environment, University of Chinese Academy of Sciences, Beijing, China; 5 CSIRO Agriculture and Food, Australia

**Keywords:** Elevated CO_2_ concentrations, Fe acquisition, grain Fe content, *Oryza sativa*, photosynthesis, *Rab6a*, small GTPase

## Abstract

Despite extensive studies on the effects of elevated atmospheric CO_2_ concentrations ([CO_2_]) on rice, the molecular mechanisms and signaling events underlying the adaptation of plants remain largely elusive. Here, we report that *OsRab6a*, which encodes a small GTPase, is involved in the regulation of rice growth, grain yield, and accumulation of iron (Fe) in response to elevated [CO_2_] (e[CO_2_]). We generated transgenic plants with *OsRab6a*-overexpression (-OE) together with *OsRab6a-*RNAi lines, and found no differences in growth and grain yield among them and wild-type (WT) plants under ambient [CO_2_] conditions. Under e[CO_2_] conditions, growth and grain yield of the WT and *OsRab6a*-OE plants were enhanced, with a greater effect being observed in the latter. In contrast, there were no effects of e[CO_2_] on growth and grain yield of the *OsRab6a*-RNAi plants. Photosynthetic rates in both the WT and *OsRab6a*-OE plants were stimulated by e[CO_2_], with the magnitude of the increase being higher in *OsRab6a*-OE plants. Fe concentrations in vegetative tissues and the grain of the WT and transgenic plants were reduced by e[CO_2_], and the magnitude of the decrease was lower in the OE plants than in the WT and RNAi plants. Genes associated with Fe acquisition in the *OsRab6a*-OE lines exhibited higher levels of expression than those in the WT and the RNAi lines under e[CO_2_]. Analysis of our data using Dunnett’s multiple comparison test suggested that OsRab6a is an important molecular regulator that underlies the adaptation of rice to e[CO_2_] by controlling photosynthesis and Fe accumulation.

## Teaser

The small GTPase OsRab6a enhances growth and grain yield in rice in response to elevated CO_2_ and helps to maintain Fe concentrations in the grain via increasing the expression of genes related to Fe acquisition.

## Introduction

Rice (*Oryza sativa*) is a major crop and a staple food that feeds almost half of the world’s population (GRiSP, 2013). Numerous studies using open-top chambers and free-air CO_2_ enrichment (FACE) facilities have demonstrated that elevated atmospheric CO_2_ concentrations (e[CO_2_]) can have a great impact on the growth and development of rice plants (Seneweera and Conroy, 1997; Kim *et al.*, 2003; Fukayama *et al.*, 2009; Kanno *et al.*, 2017). Various morphological and physiological traits have been reported to be influenced by e[CO_2_], including photosynthesis, tiller number, grain yield, and the nutritive quantity of the grain. For example, Ainsworth (2008) found that grain yield was increased in plants exposed to e[CO_2_] within a FACE facility, whilst Zhang *et al.* (2013) showed that e[CO_2_] increased yield whilst grain nitrogen accumulation was reduced, leading to a reduction in grain quality. Root growth and morphology of rice are also affected when plants are grown under FACE conditions. In addition to growth and yield, e[CO_2_] can also affect the concentrations of mineral elements in rice plants. For example, it has been reported that the concentrations of Ca, Mg, Fe, Zn, and Mn in milled grains are decreased by e[CO_2_] (Yang *et al.*, 2007; Guo *et al.*, 2015; Al-Hadeethi *et al.*, 2019).

Iron (Fe) is one of the essential mineral elements for plant growth and development, and it is also an essential nutrient for human health. Fe deficiency in plants often leads to chlorosis and reductions in crop yield and quality (Luccena and Hernandez-Apaolaza, 2017), whilst low Fe contents in food have been reported to result in anemia and affect billions of people around the world (Hell and Stephan, 2003; Lee and An, 2009). Plants have evolved two distinct strategies to mobilize and acquire Fe from soils (Kobayashi and Nishizawa, 2012). In non-graminaceous monocots and dicots, plants utilize the Strategy I mechanism to maximize Fe acquisition by reducing Fe^3+^ to Fe^2+^ thorough ferric chelate reductase with subsequent transport of Fe^2+^ across the root plasma membrane by the iron transporter IRT1 (Kobayashi and Nishizawa, 2012). Acquisition of Fe in graminaceous monocot plants is achieved via the Strategy II mechanism, phytosiderophores belonging to mugineic acid (MA) family are exuded into the soil to form phytosiderophore–Fe^3+^ complexes that are subsequently taken up by roots (Kobayashi and Nishizawa, 2012). In addition to uptake of Fe^3+^ by this route, rice plants are also capable of acquiring Fe^2+^ using the OsIRT1 and OsIRT2 transporters (Bughio *et al.*, 2002; Ishimaru *et al.*, 2006). Many molecular regulators associated with Fe acquisition have been functionally characterized in rice, including OsIDEF1, OsIDEF2, OsIRO2, OsHRZ1, OsHRZ2, and OsRMC (Ogo *et al.*, 2007, 2008; Kobayashi and Nishizawa, 2012; Kobayashi *et al.*, 2012, 2013, 2014; Yang *et al.*, 2013). These studies have provided promising clues for improving the efficiency of Fe acquisition under ambient [CO_2_]; however, few studies have investigated how the regulatory roles of these proteins in Fe homeostasis are affected under conditions of e[CO_2_].

The responses and adaptations of plants to e[CO_2_] have been well characterized at physiological level; however, much less is known about the molecular mechanisms and signaling events that underlie these adaptations (De Souza *et al.*, 2016; Becklin *et al.*, 2017). Microarray and RNA-sequencing techniques have shown that e[CO_2_] can have a great impact on gene expression in plants (De Souza *et al.*, 2016). Fukayama *et al.* (2009) demonstrated that e[CO_2_] significantly alters the expression of rice genes involved in signal transduction and transcription regulation, and they went on to show that genes involved in CO_2_ fixation are down-regulated by e[CO_2_], while genes involved in RuBP generation and starch synthesis are up-regulated (Fukayama *et al.*, 2011). In addition to studies of whole-transcriptome responses to e[CO_2_], there have also been investigations into the functioning of some specific genes involved in CO_2_-dependent physiological processes. Rubisco is a key enzyme that catalyses CO_2_ fixation in photosynthesis. Over- and underexpression of *Ribulose bisphosphate carboxylase small chain* (*RBCS*) alters whole-plant growth and N allocation under varying [CO_2_] conditions (Makino *et al.*, 2000; Sudo *et al.*, 2014), and it has also been demonstrated that antisense transgenic rice with suppression of *RBCS* can increase their photosynthetic capacity and biomass production under e[CO_2_] (Kanno *et al.*, 2017). A *CRCT* gene encoding CONSTANS, CONSTANS-like, and TOC1 (CCT) domain-containing protein has also been found to be [CO_2_]-responsive (Morita *et al.*, 2015). CRCT may be a key protein that regulates the expression of [CO_2_]-responsive genes involved in the adaptation of rice plants to e[CO_2_]. Overexpression of *CRCT* leads to an increase in starch content in rice straw, which could provide an effective genetic engineering approach for bioethanol production under future e[CO_2_] conditions (Morita *et al.*, 2017). May *et al.* (2013) identified some e[CO_2_]-responsive microRNAs in Arabidopsis using RNA-sequencing. Among these, miR156/157 and miR172 were found to be involved in the induction of early flowering under e[CO_2_]. RNA-seq has also been used to identify two key regulatory genes, *SCRM2* and *CDKB1*, that control stomatal patterning in response to e[CO_2_] in *Plantago lanceolata* (Watson-Lazowski *et al.*, 2016). These studies have provided insights that link molecular mechanisms to physiological processes in response to e[CO_2_]. However, functional characterization of more candidate genes that are involved in the mediation of CO_2_-dependent physiological processes are needed to improve our knowledge of the molecular basis for plant responses to e[CO_2_].

Genes for small GTPases that encode monomeric G proteins related to the α-subunit of heterotrimeric G proteins play important roles in cellular signal transduction in plant growth and development, and in response to environmental cues (Yang, 2002; Vernoud *et al.*, 2003; Wang *et al.*, 2013). There is evidence that small GTPases are involved in the regulation of plant responses to environmental stresses such as salt stress (Wang *et al.*, 2013). In a previous study, we identified the involvement of a rice small GTPase, OsRab6a, in the regulation of Fe homeostasis (Yang and Zhang, 2016), and found that overexpression of *OsRab6a* increased the Fe concentration in the grains under ambient [CO_2_] conditions. In the current study, we aimed to determine whether a similar OsRab6a-mediated increase in Fe occurs under e[CO_2_] conditions. We generated transgenic plants with Os*Rab6a*-overexpression together with RNAi lines and examined their responses to e[CO_2_].

## Materials and methods

### Plant material and growth conditions

Experiments were carried out on sedlings of rice (*Oryza sativa* L. ssp. *japonica*) cv. Zhonghua 10. The generation of the*OsRab6a*-overexpression (-OE) and RNAi lines has been described in detail previously by Yang and Zhang (2016). Seeds were germinated in the dark and grown in soil for 30 d. For the analysis of growth at the seedling stage under ambient and elevated [CO_2_] (e[CO_2_]), the 30-d-old seedlings were transferred to plastic boxes (25×18×14 cm length×width×depth) filled with 5.5 kg of soil mixed with a compound fertilizer. The total N, P, and K concentrations in the soil were 1.9, 0.9, and 18.7 mg g^−1^, respectively, and the exchangeable Fe was 36.3 µg g^−1^. For analysis of growth at the tillering stage and the final grain yield, the 30-d-old seedlings were transferred to plastic pots (diameter 30 cm, depth 31 cm) filled with 19 kg of soil mixed with the compound fertilizer. Both sets of seedlings were distributed among eight octagonal open-top chambers (OTCs) of 4.2 m diameter and 2.4 m height, four of which were ventilated with ambient [CO_2_] whilst the other four received elevated [CO_2_]. The OTCs were located at the Observation Station on Global Change Biology of the Institute of Zoology, Chinese Academy of Sciences, in Xiaotangshan County, Beijing, China (40^o^11´N, 116 ^o^ 24´E). The measured CO_2_ concentrations (daily mean ±SD) were 383±26 μmol mol^−1^ in the ambient chambers and 769±23 μmol mol^−1^ in the e[CO_2_] chambers. Full details of the OTCs and the automatic control system for the CO_2_ concentration have been reported previously (Chen *et al.* 2005; Sun *et al.*, 2010; Tian *et al.*, 2013).

### Measurement of photosynthetic rate

Measurements of photosynthetic rate were taken on plants after they had been in the OTCs for 20 d (seedling stage) and 60 d (tillering stage), respectively. Measurements were taken between 08.30 h and 11.30 h using a LI-6400 XT portable photosynthesis system (Li-Cor). Artificial illumination from a red-blue 6400-02B LED light source that could release continuous light at 1000 μmol m^−2^ s^−1^ photosynthetic photon flux density was used during the measurements.

### Measurements of seedling height, biomass, and Fe concentrations in the shoots, roots, and grains

The shoots and roots of the plants in the OTCs were collected separately at the seedling stage (after 20 d in the chambers) and the tillering stage (after 60 d in the chambers). After determination of plant height (to the highest leaf tip) and biomass dry weight, the shoot and root samples were ground separately to a fine powder, and digested in 6 ml of concentrated nitric acid and 2 ml of hydrogen peroxide using a MARS microwave digestion system (CEM, Buckingham, WA, USA). Grains harvested from mature plants were also dried and digested in the same way. Fe concentrations were measured by inductively coupled plasma mass spectrometry (ICAP6300; ThermoScientific).

### RNA extraction and real-time PCR

Total RNA was extracted using RNAiso reagent (Takara) and reverse-transcribed into first-strand cDNA using a PrimeScript^®^ RT Reagent kit with gDNA Eraser (Takara). Real-time PCR was conducted in an optical 96-well plate using an Applied Biosystems Stepone™ Real-Time PCR system. Each reaction contained 0.5 µl of cDNA samples, 0.6 µl of 10 µM gene-specific primers, and 7.5 µl of 2× SYBR Green Master Mix reagent in a final volume of 15 µl. The thermal cycling consisted of 95 °C for 10 min, and 40 cycles at 95 °C for 30 s, 60 °C for 30 s, and 72 °C for 30 s. The genes examined were *OsRab6a*, *OsNAS1* and *2*, which encode enzymes catalysing the synthesis of 2′-deoxymugineic acid (DMA) to chelate Fe^3+^, and *OsIRT1*, which encodes transporters of Fe^2+^ uptake. Measurements were taken after the seedlings had been in the OTCs for 10 d. The primers used are as follows: *OsRab6a*, 5´-CTTTGGGATACAGCTGGGCA -3´ and 5´-TGCCTGTCAGTCACATCGTAAA-3´; *OsNAS1*, 5´-GTCTAACAGCCGGAC GATCGAAAGG-3´ and 5´-TTTCTCACTGTCATACACAGATGGC-3´; *OsNAS2*, 5´- TGAGTGCGTGCATAGTAATCCTGGC-3´ and 5´-CAGACGG TGACA AACACCTCTTGC-3´ (Inoue *et al.*, 2003); *OsIRT1*, 5´-CGT CTTCTTC TTCTCCACCACGAC-3´ and 5´-GCAGCTGA TGATCGAGTCTGACC-3´; and *actin* (GenBank accession no. AB047313), 5´-ACCACAGGTATTGTGTTGGACTC-3´ and 5´-AG AGCATATCCTTCATAGATGGG-3´. Amplification of actin was used as an internal control. The relative expression level was determined using the comparative *C*_T_ method.

### Statistical analysis

For analysis of growth, grain yield, and photosynthesis, two replicate plants were measured in each of the chambers, and ANOVAs were carried out on the basis of a nested design. For analysis of Fe concentrations, one replicate plant in each of the chambers was measured, and for analysis of gene expression, three replicate plants in one chamber were measured. Significant differences between ambient [CO_2_] and e[CO_2_] for the same genotype were determined by Student’s *t*-test. Significant differences among WT, OE, and RNAi plants within the same [CO_2_] treatment were evaluated using ANOVA followed by Dunnett’s multiple comparison test within GraphPad Prism (https://www.graphpad.com/).

## Results

### Effects of elevated [CO_2_] on expression of *OsRab6a*

**Fig. 1.**
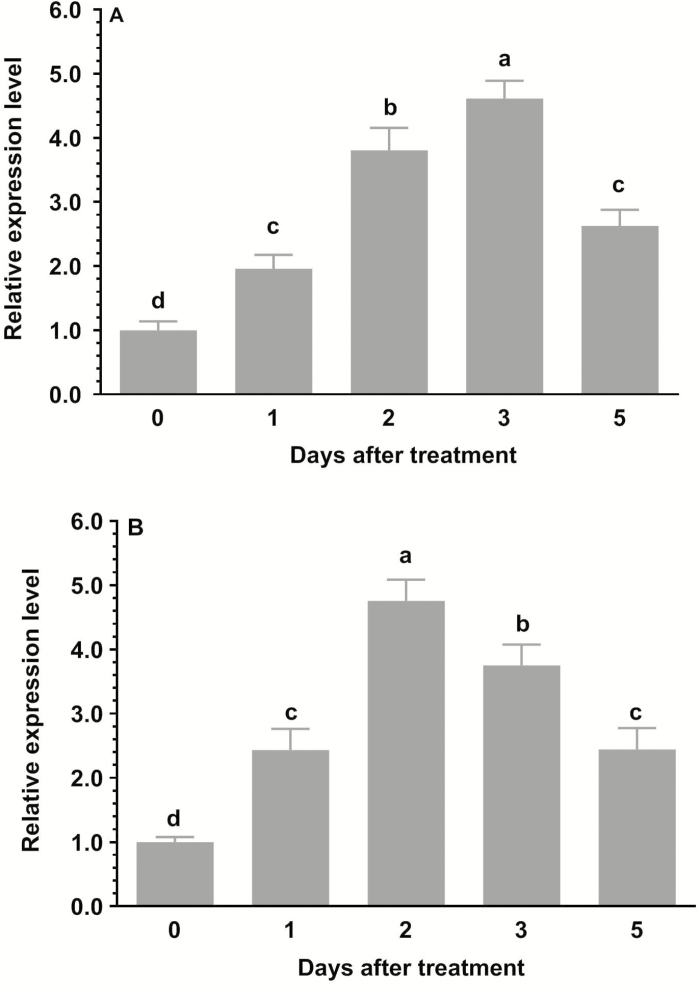
Time course of *OsRab6a* expression in rice seedlings under elevated [CO_2_] in (A) shoots and (B) roots. Wild-type seedlings of 30-d-old were placed in an open-top chamber with [CO_2_] of ~770 μmol mol^−1^ for 5 d. Expression is relative to the value before treatment (0 d), which was set as 1, and *actin* was used as the reference gene. Data are means (±SE), *n*=3. Different letters indicate significant differences at *P*<0.05 between the expression level of *OsRab6a* under ambient and elevated [CO_2_] treatments, as determined using ANOVA followed by Dunnett’s multiple comparison test (*P*<0.05)

Exposure of WT rice seedlings to e[CO_2_] led to an increase in the expression of *OsRab6a* in both shoots and roots (Fig. 1A, B). Transcript levels peaked after 3 d of treatment in the shoots and after 2 d in the roots, and declined thereafter.

### Effects of elevated [CO_2_] on plant growth

**Fig. 2.**
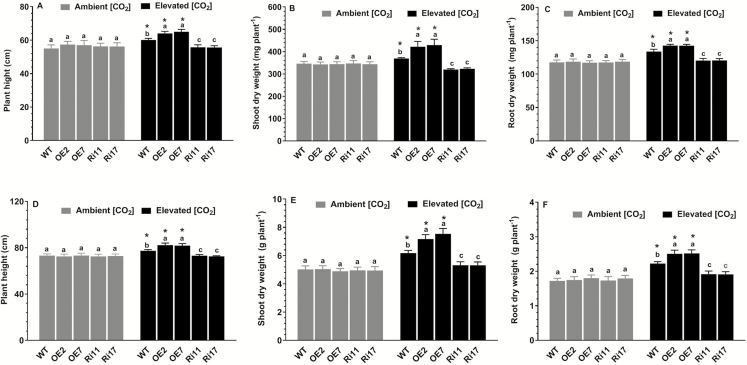
Effects of elevated [CO_2_] (e[CO_2_]) on the growth of rice wild-type (WT) seedlings and transgenic lines with *OsRab6a*-overexpression (OE) or *OsRab6a*-RNAi (Ri). Seedlings at 30 d old were placed in open-top chambers receiving either ambient [CO_2_] (~380 μmol mol^−1^) or e[CO_2_] (~770 μmol mol^−1^) and measurements were taken after 20 d (seedling stage) and after 60 d (tillering stage). (A–C) Seedling stage: (A) plant height, (B) shoot dry weight, and (C) root dry weight. (D–F) Tillering stage: (D) plant height, (E) shoot dry weight, and (F) root dry weight. Data are means (±SE), *n*=8, with two plants being sampled in each of four replicate chambers. Asterisks indicate a significant difference between ambient [CO_2_] and e[CO_2_] for a given genotype, as determined using Student’s *t*-test (*P*<0.05). Different letters indicate significant differences between the genotypes within the same [CO_2_] treatment, as determined using ANOVA followed by Dunnett’s multiple comparison test (*P*<0.05).

Given the up-regulation of *OsRab6a* by e[CO_2_] in the WT, we then examined growth in overexpression (OE) and RNAi lines. Two independent *OsRab6a*-OE lines (OE2 and OE7) and two RNAi lines (Ri11 and Ri17) were used for all subsequent experiments. Under ambient [CO_2_] conditions, there were no differences in phenotypes between the WT and transgenic plants at either the seedling or tillering stages (Fig. 2). In comparison, under e[CO_2_] both the WT and OE plants showed increases in height, and shoot and root weight compared with ambient [CO_2_], with the effects being significantly greater in the OE lines. In contrast, the *OsRab6a*-RNAi lines showed no significant responses to e[CO_2_] compared with ambient [CO_2_].

### Effects of elevated [CO_2_] on photosynthesis

**Fig. 3.**
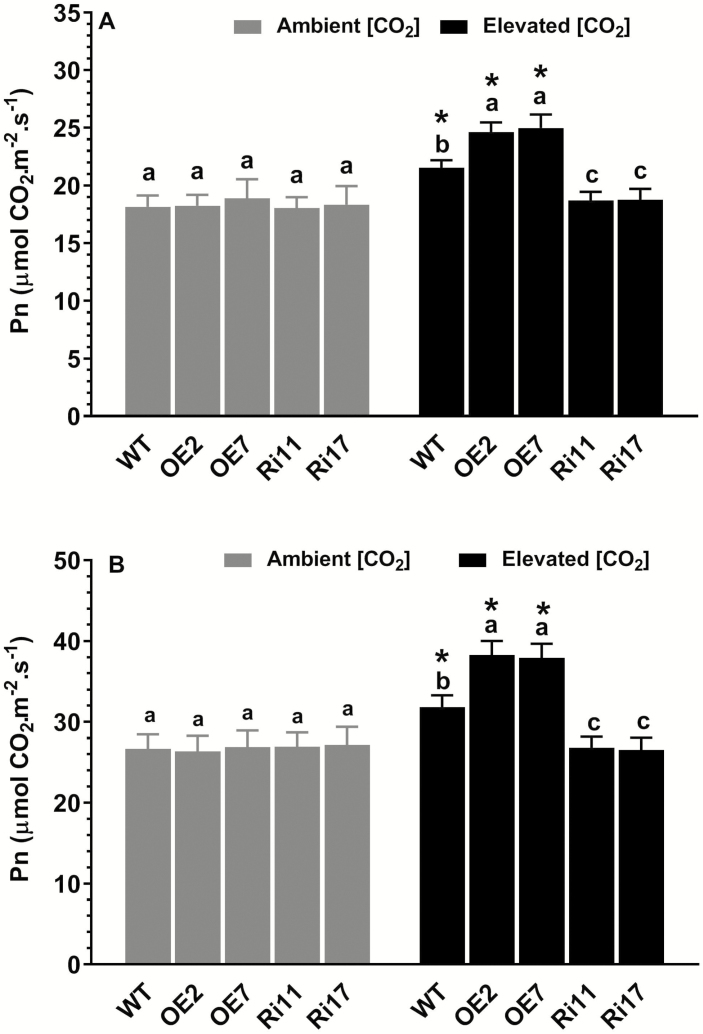
Effects of elevated [CO_2_] (e[CO_2_]) on the net rate of photosynthesis (*P*_n_) in rice wild-type (WT) seedlings and transgenic lines with *OsRab6a*-overexpression (OE) or *OsRab6a*-RNAi (Ri). Seedlings of 30-d-old were placed in open-top chambers receiving either ambient [CO_2_] (~380 μmol mol^−1^) or e[CO_2_] (~770 μmol mol^−1^) and measurements were taken (A) after 20 d (seedling stage) and (B) after 60 d (tillering stage). Data are means (±SE) *n*=8, with two plants being sampled in each of four replicate chambers. Asterisks indicate a significant difference between ambient [CO_2_] and e[CO_2_] for a given genotype, as determined using Student’s *t*-test (*P*<0.05). Different letters indicate significant differences between the genotypes within the same [CO_2_] treatment, as determined using ANOVA followed by Dunnett’s multiple comparison test (*P*<0.05).

No differences in photosynthetic rates were observed among the WT, OE, and RNAi plants under ambient [CO_2_] at either the seedling or tillering stages (Fig. 3). The photosynthetic rates of the WT and OE plants were significantly enhanced by e[CO_2_], with the magnitude of enhancement being greater in *OsRab6a*-OE. In contrast, the photosynthetic rates of RNAi plants were not responsive to e[CO_2_].

### Effects of elevated [CO_2_] on grain yield

**Fig. 4.**
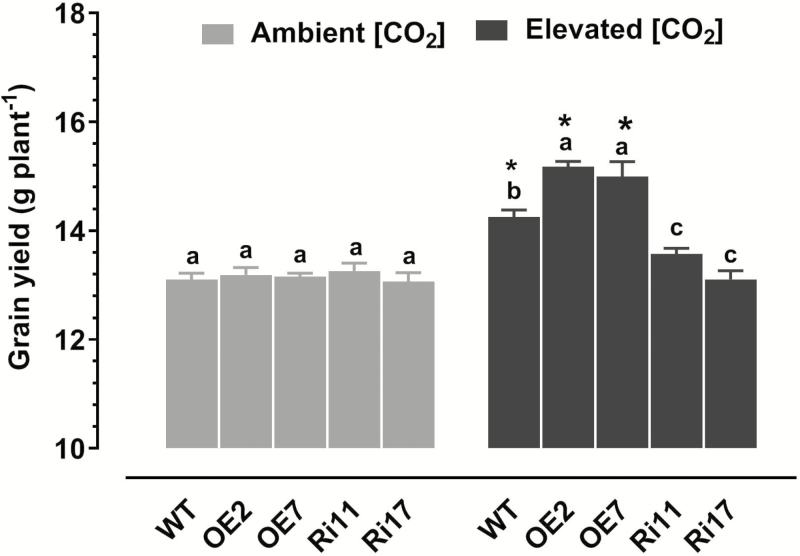
Effects of elevated [CO_2_] (e[CO_2_]) on final grain yield in rice wild-type (WT) seedlings and transgenic lines with *OsRab6a*-overexpression (OE) or *OsRab6a*-RNAi (Ri). Seedlings of 30-d-old were placed in open-top chambers receiving either ambient [CO_2_] (~380 μmol mol^−1^) or e[CO_2_] (~770 μmol mol^−1^) and grown to maturity. Data are means (±SE), *n*=8, with two plants being sampled in each of four replicate chambers. Asterisks indicate a significant difference between ambient [CO_2_] and e[CO_2_] for a given genotype, as determined using Student’s *t*-test (*P*<0.05). Different letters indicate significant differences between the genotypes within the same [CO_2_] treatment, as determined using ANOVA followed by Dunnett’s multiple comparison test (*P*<0.05).

There were no differences in grain yield between the WT and the transgenic plants under ambient [CO_2_] (Fig. 4). Elevated [CO_2_] increased the grain yield of both the WT and OE plants, and again the effect was significantly greater in the OE plants. The grain yield of the RNAi plants was not altered by e[CO_2_].

### Effects of elevated [CO_2_] on tissue Fe concentrations

**Fig. 5.**
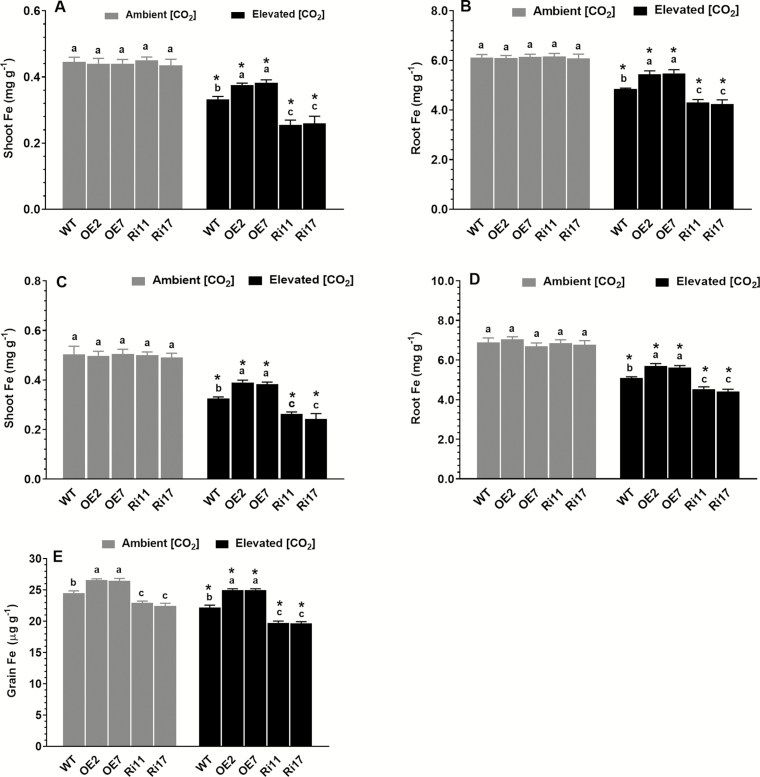
Effects of elevated [CO_2_] (e[CO_2_]) on Fe concentrations in rice wild-type (WT) seedlings and transgenic lines with *OsRab6a*-overexpression (OE) or *OsRab6a*-RNAi (Ri). Seedlings at 30-d-old were placed in open-top chambers receiving either ambient [CO_2_] (~380 μmol mol^−1^) or e[CO_2_] (~770 μmol mol^−1^) and measurements were taken (A) after 20 d (seedling stage) and (B) after 60 d (tillering stage). (A, B) Seedling stage: Fe concentrations in (A) shoots and (B) roots. (C, D) Tillering stage: Fe concentrations in (C) shoots and (D) roots. (E) Fe concentrations in grains. Data are means (±SE), with one plant in each of four replicate chambers being measured. Asterisks indicate a significant difference between ambient [CO_2_] and e[CO_2_] for a given genotype, as determined using Student’s *t*-test (*P*<0.05). Different letters indicate significant differences between the genotypes within the same [CO_2_] treatment, as determined using ANOVA followed by Dunnett’s multiple comparison test (*P*<0.05).

OsRab6a is involved in the regulation of Fe homeostasis in rice plants under ambient [CO_2_] conditions (Yang and Zhang, 2016), and we therefore examined the effects of e[CO_2_] on Fe concentrations in the shoots, roots, and grains of the WT and transgenic plants. No significant differences in Fe concentrations in the shoots and roots among the different genotypes were observed at either the seedling or tillering stages for plants grown under ambient [CO_2_] conditions (Fig. 5A–D). In contrast, Fe concentrations in the shoots and roots of all the genotypes were significantly reduced when they were grown under e[CO_2_] compared to ambient [CO_2_] conditions. Compared with the WT, the Fe concentrations in the shoots and roots at both growth stages were higher in the OE lines and lower in the RNAi lines. Fe concentrations in the grains showed a different pattern, with a positive correlation with the *OsRab6a* expression level being observed under ambient [CO_2_] conditions (Fig. 5E). The concentrations in all the genotypes were reduced under e[CO_2_] compared with ambient [CO_2_] conditions, with the greatest reduction occurring in the RNAi lines and the smallest occurring in the OE lines.

### Effects of elevated [CO_2_] on expression of Fe-responsive genes

**Fig. 6.**
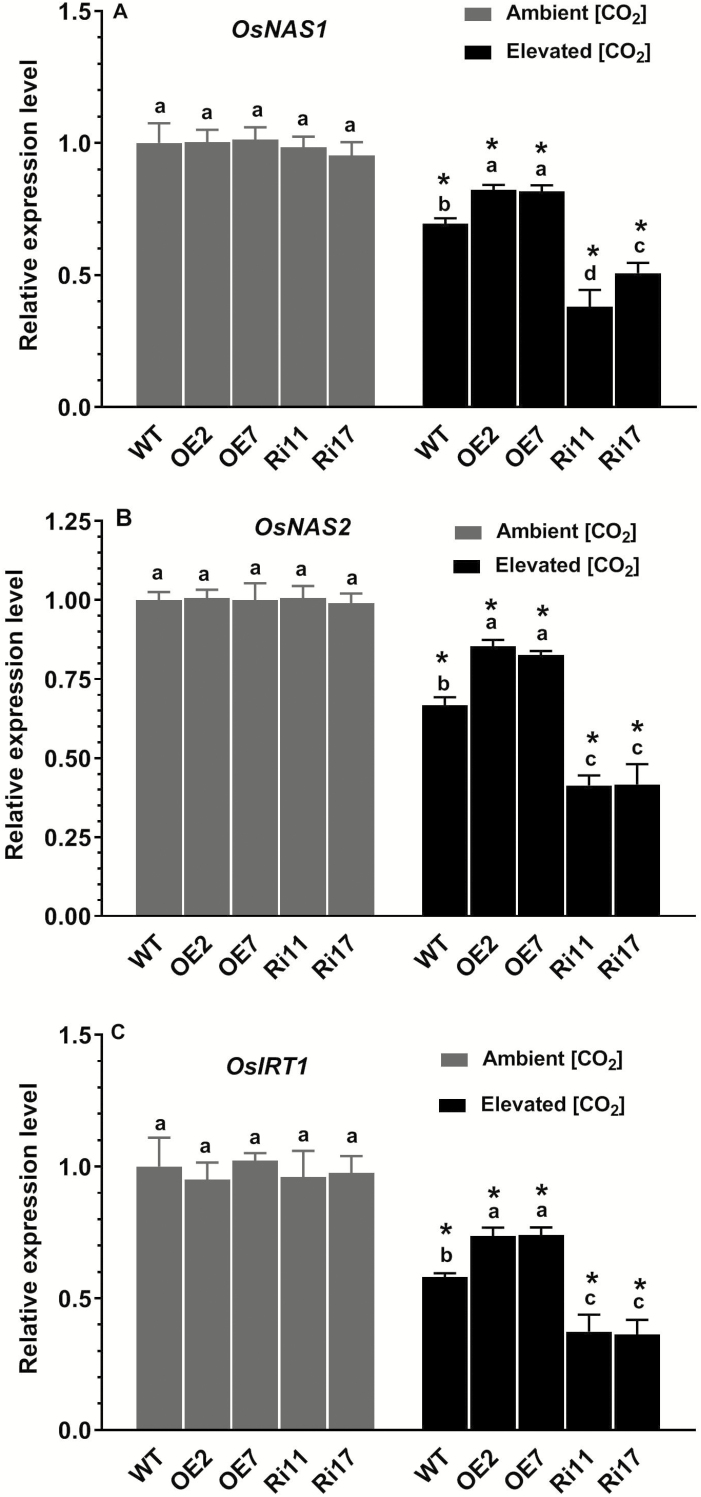
Effects of elevated [CO_2_] (e[CO_2_]) on expression of (A) *OsNAS1*, (B) *OsNAS2*, and (C) *OsIRT1* in rice wild-type (WT) seedlings and transgenic lines with *OsRab6a*-overexpression (OE) or *OsRab6a*-RNAi (Ri). Seedlings of 30-d-old were placed in an open-top chamber receiving either ambient [CO_2_] (~380 μmol mol^−1^) or e[CO_2_] (~770 μmol mol^−1^) and root samples were taken after 10 d. Expression is relative to the value in the WT under ambient [CO_2_], which was set as 1, and *actin* was used as the reference gene. Data are means (±SE), *n*=3. Asterisks indicate a significant difference between ambient [CO_2_] and e[CO_2_] for a given genotype, as determined using Student’s *t*-test (*P*<0.05). Different letters indicate significant differences between the genotypes within the same [CO_2_] treatment, as determined using ANOVA followed by Dunnett’s multiple comparison test (*P*<0.05).

We examined changes at the transcriptional level in the expression of genes involved in Fe homeostasis. *OsNAS1* and *OsNAS2* encode enzymes that catalyse the synthesis of DMA to chelate Fe^3+^, and *OsIRT1* encodes transporters of Fe^2+^ uptake (Bughio *et al.*, 2002; Inoue *et al.*, 2003; Lee and An, 2009; Kawakami and Bhullar, 2018). Consistent with the observed decreases in Fe concentrations, the expression levels of all these genes in both the WT and transgenic lines were significantly down-regulated by e[CO_2_] (Fig. 6). Furthermore, the pattern of expression was also consistent with the Fe concentration results, with higher levels in the OE lines and lower levels in the RNAi lines compared with the WT.

## Discussion

Numerous studies have demonstrated that elevated atmospheric CO_2_ concentrations (e[CO_2_]) can influence grain yield and quality in rice plants (e.g. Fukayama *et al.*, 2011); however, few studies have focused on the molecular mechanisms that underlie the responses and adaptations to e[CO_2_], particularly with regard to how e[CO_2_] modulates the nutritional quality of the grain. In the present study, we determined that a small GTPase protein, OsRab6a, plays a positive role in the regulation of rice growth and grain yield in response to e[CO_2_], such that overexpression of *OsRab6a* resulted in higher plant biomass and increased grain yield when grown in e[CO_2_] (Figs 2, 4). More importantly, our results indicated that OsRab6a also contributed to higher Fe accumulation in the grain (Fig. 5). Hence, OsRab6a has great potential for improving gain yield and nutritional quality under predicted increased future levels of atmospheric CO_2_. The increases in grain yield observed as a result of overexpressing *OsRab6a* under e[CO_2_] conditions could be accounted for by enhanced of photosynthetic rates in the transgenic plants (Fig. 3), whilst the enrichment of Fe in the grain in *OsRab6a*-overexpression (-OE) plants could be accounted for by the up-regulation of genes involved in Fe mobilization, uptake, and translocation (Fig. 6).

Small GTPases can be divided into at least five subfamilies, namely Ras, Rho, Rab, Arf, and Ran (Takai *et al.*, 2001; Yang, 2002) and they play important roles in sensing and responding to environmental cues in plants (Wang *et al.*, 2013). To the best of our knowledge, our results are the first to show the involvement of a small GTPase in the regulation of plant growth and the acquisition of mineral nutrients in rice grain in an e[CO_2_] environment. CCRE1/2/3 *cis*-elements (TGACGT, ACGTCA, and TGACGC) are CO_2_-responsive elements that were first identified by loss-of-function assays in the marine diatom *Phaeodactylum tricornutum* (Tanaka *et al.*, 2016). By examining the promoter sequence of *OsRab6a*, we discovered a CO_2_-responsive element, CCRE2 (ACGTCA) (Supplementary Fig. S1 at *JXB* online). The presence of this *cis*-element may account for the e[CO_2_]-dependent expression patterns of *OsRab6a*.

The current ambient CO_2_ concentration is below the CO_2_-saturated concentration of Rubisco in C_3_ plants (Kimball, 1983; Long *et al.*, 2006; Ainsworth, 2008). Therefore, increases in [CO_2_] will have a positive impact on photosynthetic rates, and hence contribute to increases in shoot and root growth, and in grain yield in C_3_ crop plants such as rice and wheat (Kim *et al.*, 2003; Ainsworth, 2008; Yang *et al.*, 2009, 2010). Here, we found that photosynthetic rates in both the wild-type (WT) and *OsRab6a*-OE lines were enhanced under e[CO_2_] conditions at the seedling and tillering stages, with the magnitude of the increase being greater in the OE plants (Fig. 3). This may have accounted for the corresponding increases in shoot and root biomass and in grain yield that were observed in these plants (Figs 2, 4). Photosynthesis in rice is regulated by leaf physiological parameters, such as the leaf N concentration and stomatal conductance (Ikawa *et al.*, 2018), and examination of these parameters for their involvement in the OsRab6a-mediated changes that we observed would be of interest. In addition, sink capacity has been suggested to be an important trait associated with grain yield in some rice cultivars (Nakano *et al.*, 2017). Our future research will focus on the effects of OsRab6a on sink capacity under ambient and e[CO_2_] conditions.

Iron is an essential nutrient element for human health, and it has been reported that e[CO_2_] decreases the concentration of iron in rice grains, threatening human nutrition in those Asian countries where rice is a staple food (Loladze, 2014; Myers *et al.*, 2014; Zhu *et al.*, 2018). This decline in Fe concentration has usually been attributed to a dilution effect as the result of greater accumulation of carbohydrates in grains under e[CO_2_] (Loladze, 2014; Myers *et al.*, 2014; Zhu *et al.*, 2018). Here, we found that Fe concentrations in the grains of all the genotypes were reduced by e[CO_2_], but the magnitude of the decrease was significantly less in the *OsRab6a*-OE lines than in the WT and RNAi lines (Fig. 5E). These results suggest that OsRab6a might mitigate the decline in Fe concentration that usually accompanies the increase in grain weight under e[CO_2_].

It has been reported that Fe concentrations in some vegetative organs of rice are also reduced by e[CO_2_] (Seneweera and Conroy, 1997; Johnson, 2013; Ujiie *et al.*, 2019). For example, Ujiie *et al.* (2019) found that concentrations in leaf blades were reduced under FACE conditions compared to ambient [CO_2_]. Consistent with the results reported by Seneweera and Conroy (1997), we found that e[CO_2_] decreased the Fe concentrations in both roots and shoots of the WT and transgenic plants at the seedling and tillering stages compared to ambient [CO_2_] (Fig. 5A–D). However, there have been some inconsistent results with regard to changes in Fe concentrations in rice vegetative tissues under e[CO_2_]. For example, Guo *et al.* (2015) reported that concentrations were increased in the panicles and stems of plants under e[CO_2_], whilst Li *et al.* (2017) found that accumulation of Fe in rice panicles was also enhanced under e[CO_2_] conditions using a FACE system. It is possible that the different growing environments between the studies may account for the differences in the results. For example, in our study and that of Seneweera and Conroy (1997), plants were grown in pots (in open-top chambers and growth chambers, respectively) and it is possible that root growth may have been restricted by the limited soil volume. In contrast, both Guo *et al.* (2015) and Li *et al.* (2017) conducted their experiments under FACE conditions in field where the growth of the roots would not have been restricted, thus potentially allowing the plants to acquire more nutrients.

Rice plants secrete DMA to solubilize Fe^3+^ cations, thus enabling their acquisition from the rhizosphere (Kobayashi and Nishizawa, 2012; Kawakami and Bhullar, 2018). The enzyme nicotianamine synthase (NAS), encoded by *OsNAS1*, *2*, and *3*, is responsible for the first step of the biosynthesis of DMA (Inoue *et al.*, 2003; Johnson *et al.*, 2011), whilst the ZIP (Zinc-regulated transporters, Iron-regulated transporter-like Protein) transporter OsIRT1 is involved in the uptake of Fe^2+^ (Bughio *et al.*, 2002; Lee and An, 2009; Kawakami and Bhullar, 2018). Here, we found that the expression levels of *OsNAS1*, *OsNAS2*, and *OsIRT1* were decreased by e[CO_2_] (Fig. 6); however, the expression levels of these genes were higher in the OE lines than in the WT. This suggests that these genes may account for the higher Fe concentrations in the tissues of the *OsRab6a*-OE under e[CO_2_] compared with the WT and RNAi plants.

Plants often alter the morphological traits of their roots to enhance Fe acquisition (Guerinot and Yi, 1994; López-Bucio *et al.*, 2003), and we observed that overexpression of *OsRab6a* resulted in greater root biomass under e[CO_2_] conditions compared with the WT RNAi lines (Fig. 2C, F). Hence, the increased concentrations of Fe in the OE lines may have resulted from this increase in root growth.

## Conclusions

Our results indicate that OsRab6a as an important molecular regulator involved in the growth and grain yield of rice under elevated atmospheric [CO_2_] via its modulation of the rate of photosynthesis. More importantly, we found that OsRab6a plays a regulatory role in maintaining Fe homeostasis under both ambient and elevated [CO_2_], which potentially makes it a valuable tool for biofortifying the Fe content in rice grains under future climate scenarios.

## Supplementary data

Supplementary data are available at *JXB* online.

Fig. S1. A CCRE2 *cis*-element exists in the promoter of *OsRab6a*.

eraa279_suppl_Supplementary_Figure-S1
