# Supplementary figures and images for: A rice small GTPase, Rab6a, is involved in the regulation of grain yield and iron nutrition in response to CO2 enrichment
Source: J Exp Bot. 2020 Jun 11;71(18):5680–8. doi: 10.1093/jxb/eraa279 (PMC7501819; doi:10.1093/jxb/eraa279)

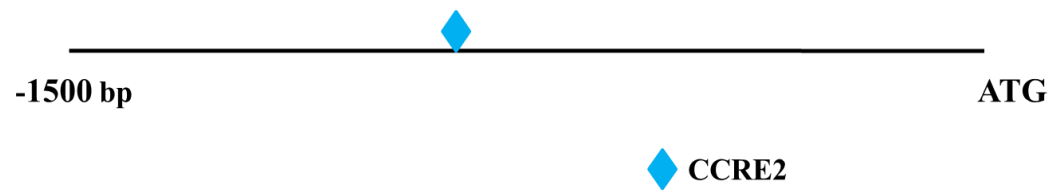

Supplemental Figure 1. A CCRE2 *cis*-element exists in the promoter of OsRab6a.

Supplement: eraa279_suppl_Supplementary_Figure-S1 [file eraa279_suppl_supplementary_figure-s1.pdf]
